# Supplementary material for: A prospective study to explore the relationship between MTHFR C677T genotype, physiological folate levels, and postpartum psychopathology in at-risk women
Source: PLoS One. 2020 Dec 14;15(12):e0243936. doi: 10.1371/journal.pone.0243936 (PMC7735580; doi:10.1371/journal.pone.0243936)
Supplement: S1 Table — (DOCX) [file pone.0243936.s001.docx]

**S1 Table**

Regression coefficients and 95%CI from linear regressions (beta) and logistic regression (OR) for the three psychological outcome scores. Coefficients for the interaction terms are only shown for the CARS-M model. RBC folate was divided by 100 to make the coefficients interpretable.

|  | Outcome variable | | |
| --- | --- | --- | --- |
|  | EPDS | log(CARS-M) | PANSS |
| Variables: | beta (95%CI) | beta (95%CI) | OR (95%CI) |
| Intercept | 10.25 (8.50 to 11.99) | 2.00 (1.71 to 2.30) | - |
| *MTHFR* CT^1^ | -0.11 (-1.39 to 1.17) | 0.25 (-0.24 to 0.73) | 1.16 (0.66 to 2.03) |
| *MTHFR* *TT*^1^ | 0.98 (-1.10 to 3.07) | -0.28 (-0.82 to 0.25) | 1.12 (0.44 to 2.61) |
| RBC folate | -0.20 (-0.45 to 0.05) | -0.04 (-0.08 to 0.00) | 0.90 (0.80 to 1.02) |
| *MTHFR* CTxRBC folate | - | -0.02 (-0.09 to 0.06) | - |
| *MTHFR* *TT*xRBC folate | - | 0.06 (0.01 to 0.12) | - |

^1^reference = *MTHFR* genotype *CC*

*EPDS*:

The intercept is the predicted value of EPDS when *MTHFR* = CC and RBC folate = 0. For every increase in 100 units of RBC folate, the EPDS score goes down by 0.2 points regardless of genotype. If the *MTHFR* genotype = CT, the average EPDS score is reduced by 0.11 relative to CC (the whole line is lower), and for TT it’s increased by 0.98 points relative to CC. None of these coefficients are significant.

*CARS-M:*

The intercept is the predicted log(CARS-M) when *MTHFR* = CC and RBC folate = 0. The RBC folate coefficient says that for CC genotype, the log(CARS-M) score decreases by 0.04 for every increase in 100 units of RBC folate. Now it gets a bit trickier for the other genotypes. For CT, log(CARS-M) at the intercept (when RCB folate = 0) is 0.25 higher than for CC, AND the slope for RBC folate is 0.02 lower. This means that for every increase in 100 units of RBC folate, we expect a decrease of -0.04-0.02 = -0.06 in log(CARS-M) score. FOR TT, log(CARS-M) at the intercept is 0.28 lower than CC, and the slope is 0.06 higher. For every increase in 100 units of RBC folate, we expect -0.04 + 0.06 = 0.02 increase in log(CARS-M) score.

*PANSS*

None of the coefficients are significant, but they would be interpreted as follows. For *MTHFR* CT genotype, the odds of meeting criteria are 16% higher than the odds for the CC genotype (but the 95%CI range from 44% lower to 2 times higher). For *MTHFR* TT the odds of meeting criteria are 12% higher than for CC. Finally, for every increase in 100 units of RBC folate, the odds of meeting criteria decrease by 10%.
