# Supplementary material for: A prospective study to explore the relationship between MTHFR C677T genotype, physiological folate levels, and postpartum psychopathology in at-risk women
Source: PLoS One. 2020 Dec 14;15(12):e0243936. doi: 10.1371/journal.pone.0243936 (PMC7735580; doi:10.1371/journal.pone.0243936)
Supplement: Attachment — Submitted filename: Report to the Authors.pdf [file pone.0243936.s002.pdf]

Report to the Authors

**A prospective study to explore the relationship between MTHFRC677T genotype, physiological folate levels, and postpartum psychopathology in at-risk women**
Manuscript: PONE-D-20-16875

The Authors analyse the relation between MTHFR C677T genotype, folate levels, and postpartum psychopathology in at-risk women. The Authors hypothesise that, in the first three months postpartum, compared to MTHFR CC homozygous women, TT homozygous women would have increased symptoms of postpartum depression and that this relationship would be moderated by physiological levels of red blood cell (RBC) folate. In addition, they conduct an exploratory analysis to assess the impact of *MTHFR C677T* genotypes and RBC folate levels on postpartum mania and postpartum psychosis.

The paper could benefit from clarification about data as well as the methodologies used. The Authors should provide more details on the modelling choices to help readers in having a clear understanding on the proposed study. Some important issues are listed below.

1. The Authors conducted a longitudinal observational study on a sample of 365 pregnant women with a history of a mood or psychotic disorder and that data collection occurred at 4 timepoints. However, the longitudinal aspect of this study is not exploited in the analysis where only the highest postpartum EPDS and CARS-M scores (from all the available postpartum timepoints) and the corresponding RBC folate levels were selected. Along the same line, RBC folate levels corresponding to the first postpartum timepoint for which the participant met criteria for psychosis, or to the earliest time point for participants which never met criteria for psychosis were chosen. All these choices might be due to a high presence of missing values at different time points but this is not clear from the text, where the only reason provided is linked to differences in timing for the emergence of symptoms of depression, mania and psychosis. The Authors should discuss in more detail why they do not perform a longitudinal statistical analysis.

2. To assess the hypothesised relationships, the Authors run a moderation analysis but they do not include any references to the literature on the moderation model. They should provide a clear and rigorous description of the moderation model implemented.

3. The Authors affirm that there are outliers in the data but they do not specify if the outliers affect only the dependent variables or also the folate levels, and neither do they provide statistical results to support their claim

4. To evaluate the relationships included in the moderation model, the Authors use robust linear regression. At line 192, they refer to “minimum maximum likelihood estimation”, which to the best of my knowledge does not exist. They are probably referring to the MM-estimator introduced by

Yohai (1987). The Author should provide references for this method and specify the software used to perform the analysis.

Yohai, J. V. (1987). High Breakdown-Point and High Efficiency Robust Estimates for Regression, Annals of Statistics, 17, 4, 1662-1683.

5. How were post-hoc analyses conducted? Methodological aspect should be detailed in the manuscript.

6. I would suggest to move Table 1: Demographic information for each MTHFR C677T genotype, into the Supplementary data, and to include the results provided in Supplementary Table 1 in the manuscript, along with p-values, which are not reported in this Table.

7. In the Discussion, the Authors affirm that an inverse relationship between folate and depressive symptoms may exist in the postpartum for women with a *MTHFR C677T* 341 *C*. However, this conclusion is not supported by the analysis.

8. At lines 372-375, it reads "While there was no statistical difference in RBC folate levels […] between CC, CT, and TT genotypes,". p-values reported in Tables 2 (p=0.08), 3 (p=0.004) and 4 (p=0.002) do not support this conclusion. This has to be clarified.

9. In the Limitation section, the Authors state that "participants with a TT genotype may, by chance, have been taking a greater amount of folic acid" (line 379). Could it be worth of investigation the inclusion of covariates, related to whether participants were taking a Folic Acid Supplement or a daily psychotropic medication, into the model?
