# Supplementary material for: A prospective study to explore the relationship between MTHFR C677T genotype, physiological folate levels, and postpartum psychopathology in at-risk women
Source: PLoS One. 2020 Dec 14;15(12):e0243936. doi: 10.1371/journal.pone.0243936 (PMC7735580; doi:10.1371/journal.pone.0243936)
Supplement: Attachment — Submitted filename: Response to Reviewers_oct7.docx [file pone.0243936.s003.docx]

***We thank the reviewers for the comments and feedback on our manuscript. We have documented our responses below (in bold italics) and have made the necessary changes to the manuscript.***

1. Please ensure that your manuscript meets PLOS ONE's style requirements, including those for file naming. The PLOS ONE style templates can be found at

https://journals.plos.org/plosone/s/file?id=wjVg/PLOSOne_formatting_sample_main_body.pdf and

<https://journals.plos.org/plosone/s/file?id=ba62/PLOSOne_formatting_sample_title_authors_affiliations.pdf>

***Thank you. Changes have been made to reflect the formatting style.***

2. Thank you for stating the following in the Acknowledgments Section of your manuscript:

"This work was made possible through funding from the Canadian Institutes of
 Health Research (CIHR), JA was supported by BC Mental Health and Substance Use
Services, the Canada Research Chairs Program, the Michael Smith Foundation for Health
Research, and CIHR."

We note that you have provided funding information that is not currently declared in your Funding Statement. However, funding information should not appear in the Acknowledgments section or other areas of your manuscript. We will only publish funding information present in the Funding Statement section of the online submission form.

Please remove any funding-related text from the manuscript and let us know how you would like to update your Funding Statement. Currently, your Funding Statement reads as follows:

"JA received funding from the Canadian Institutes of Health Research (https://cihr-irsc.gc.ca/e/193.html) to conduct this work (CIHR MOP 82750). The funders had no role in study design, data collection and analysis, decision to publish, or preparation of the manuscript."

***Thank you for bringing this to our attention. We have deleted this text from our Acknowledgements. Please update our funding statement to say:***

***This work was made possible through funding from the Canadian Institutes of
 Health Research (CIHR), JA was supported by BC Mental Health and Substance Use
Services, the Canada Research Chairs Program, the Michael Smith Foundation for Health
Research, and CIHR. JA received funding from the Canadian Institutes of Health Research (https://cihr-irsc.gc.ca/e/193.html) to conduct this work (CIHR MOP 82750). The funders had no role in study design, data collection and analysis, decision to publish, or preparation of the manuscript*.**

3. We note that you have indicated that data from this study are available upon request. PLOS only allows data to be available upon request if there are legal or ethical restrictions on sharing data publicly. For more information on unacceptable data access restrictions, please see http://journals.plos.org/plosone/s/data-availability#loc-unacceptable-data-access-restrictions.

In your revised cover letter, please address the following prompts:

a) If there are ethical or legal restrictions on sharing a de-identified data set, please explain them in detail (e.g., data contain potentially sensitive information, data are owned by a third-party organization, etc.) and who has imposed them (e.g., an ethics committee). Please also provide contact information for a data access committee, ethics committee, or other institutional body to which data requests may be sent.

***Our data contains potentially sensitive information. Further, in order to share de-identified data, our Research Ethics Board requires that participants explicitly consent. Unfortunately this was not included in our consent form for this study and we are unable to provide a de-identified data set to be made accessible. Data access requests can be sent to the University of British Columbia Clinical Research Ethics board (ethics.research.ubc.ca).***

b) If there are no restrictions, please upload the minimal anonymized data set necessary to replicate your study findings as either Supporting Information files or to a stable, public repository and provide us with the relevant URLs, DOIs, or accession numbers. For a list of acceptable repositories, please see http://journals.plos.org/plosone/s/data-availability#loc-recommended-repositories.

We will update your Data Availability statement on your behalf to reflect the information you provide.

4. Please include captions for your Supporting Information files at the end of your manuscript, and update any in-text citations to match accordingly. Please see our Supporting Information guidelines for more information: [http://journals.plos.org/plosone/s/supporting-information](about:blank).

***Thank you for bringing this to our attention. We have included these captions in the manuscript and updated our in-text citations.***

 5. Review Comments to the Author

Please use the space provided to explain your answers to the questions above. You may also include additional comments for the author, including concerns about dual publication, research ethics, or publication ethics. (Please upload your review as an attachment if it exceeds 20,000 characters)

Reviewer #1: The Authors analyse the relation between MTHFR C677T genotype, folate levels, and postpartum psychopathology in at-risk women. The Authors hypothesise that, in the first three months postpartum, compared to MTHFR CC homozygous women, TT homozygous women would have increased symptoms of postpartum depression and that this relationship would be moderated by physiological levels of red blood cell (RBC) folate. In addition, they conduct an exploratory analysis to assess the impact of MTHFR C677T genotypes and RBC folate levels on postpartum mania and postpartum psychosis.
The paper could benefit from clarification about data as well as the methodologies used. The Authors should provide more details on the modelling choices to help readers in having a clear understanding on the proposed study. Some important issues are listed below.

1. The Authors conducted a longitudinal observational study on a sample of 365 pregnant women with a history of a mood or psychotic disorder and that data collection occurred at 4 timepoints. However, the longitudinal aspect of this study is not exploited in the analysis where only the highest postpartum EPDS and CARS-M scores (from all the available postpartum timepoints) and the corresponding RBC folate levels were selected. Along the same line, RBC folate levels corresponding to the first postpartum timepoint for which the participant met criteria for psychosis, or to the earliest time point for participants which never met criteria for psychosis were chosen. All these choices might be due to a high presence of missing values at different time points but this is not clear from the text, where the only reason provided is linked to differences in timing for the emergence of symptoms of depression, mania and psychosis. The Authors should discuss in more detail why they do not perform a longitudinal statistical analysis.

***We appreciate this comment and the opportunity to provide further clarity in the manuscript. Indeed, as the reviewer mentions, there are some missing values, resulting in not all participants having data at each timepoint, limiting the number of participants who have data across all timepoints. The focus of our study; however, was not on the longitudinal evolution of psychiatric symptoms but, rather to observe the most severe symptoms (depression and mania) or if they every met criteria (psychosis), in our study time frame to test differences between genotypes. We have added the limitation of missing data to our limitation section to reflect how this may have influenced our findings.***

2. To assess the hypothesised relationships, the Authors run a moderation analysis but they do not include any references to the literature on the moderation model. They should provide a clear and rigorous description of the moderation model implemented.

***We have modified the analysis section of our manuscript to provide more clarity on our model and have included references where necessary.***

3. The Authors affirm that there are outliers in the data but they do not specify if the outliers affect only the dependent variables or also the folate levels, and neither do they provide statistical results to support their claim

***We thank the reviewer for highlighting this – we have added further details to our analysis section to better explain our analyses of outliers; specifically that their presence was assessed using diagnostic regression plots of non-linear models.***

4. To evaluate the relationships included in the moderation model, the Authors use robust linear regression. At line 192, they refer to “minimum maximum likelihood estimation”, which to the best of my knowledge does not exist. They are probably referring to the MM-estimator introduced by Yohai (1987). The Author should provide references for this method and specify the software used to perform the analysis.
Yohai, J. V. (1987). High Breakdown-Point and High Efficiency Robust Estimates for Regression, Annals of Statistics, 17, 4, 1662-1683.

***The reviewer is correct and we have revised the manuscript and have have added appropriate references regarding our use of MM-estimation to mitigate the effects of outliers and highly influential points.***

5. How were post-hoc analyses conducted? Methodological aspect should be detailed in the manuscript.

***We have provided more specific details about our “post-hoc” analysis in the manuscript.***

6. I would suggest to move Table 1: Demographic information for each MTHFR C677T genotype, into the Supplementary data, and to include the results provided in Supplementary Table 1 in the manuscript, along with p-values, which are not reported in this Table.

***We thank the reviewers for this suggestion. The p-values associated with the Supplementary data appear in the main body of the manuscript. As table 1 contains information fundamental to interpretation of the data, we have left this in the main body of the manuscript.***

7. In the Discussion, the Authors affirm that an inverse relationship between folate and depressive symptoms may exist in the postpartum for women with a MTHFR C677T 341 C. However, this conclusion is not supported by the analysis.

***We appreciate the opportunity to clarify this for the reviewer. The reviewer is correct, there is no relationship. We have edited our previously ambiguous wording to make this clearer in the manuscript.***

8. At lines 372-375, it reads “While there was no statistical difference in RBC folate levels […] between CC, CT, and TT genotypes,”. p-values reported in Tables 2 (p=0.08), 3 (p=0.004) and 4 (p=0.002) do not support this conclusion. This has to be clarified.

***We thank the reviewer for highlighting this discrepancy. This statement in the limitations section was an oversight on our part. We have edited it to more accurately reflect our data.***

9. In the Limitation section, the Authors state that “participants with a TT genotype may, by chance, have been taking a greater amount of folic acid” (line 379). Could it be worth of investigation the inclusion of covariates, related to whether participants were taking a Folic Acid Supplement or a daily psychotropic medication, into the model?

***We thank the reviewer for this comment. We have included whether participants were taking a folic acid supplement, and psychotropic medication in Table 1 and compared against genotype groups, for which there were no significant differences - with the majority of individuals taking a folic acid supplement, and a minority taking medications for all genotype groups.***

Reviewer #2: The manuscript entitled “A prospective study to explore the relationship between MTHFRC677T genotype, physiological folate levels, and postpartum psychopathology in at-risk women” is an interesting and well structured study investigating the influence of MTHFR C677T polymorphism and folate blood levels on the onset of psychopathological disorders in the postpartum. The study design is methodologically flawless and statistical data analysis is appropriate and properly conducted. The graphs are immediately explanatory. It is also written in fluent and clear English. The topic is interesting for a wide audience.

An increase in homocysteine levels resulting from folate deficiency and / or MTHFR mutations, has also been described as a possible risk factor for neuropsychiatric disorders, as well as for various other pathological situations, such as cardiovascular disease and osteoporosis, probably facilitating an increase in inflammatory factors, as suggested in a recent paper, to which the Authors could possibly refer (De Martinis M, Sirufo MM, Nocelli C, Fontanella L, Ginaldi L. Hyperhomocysteinemia is Associated with Inflammation, Bone Resorption, Vitamin B12 and Folate Deficiency and MTHFR C677T Polymorphism in Postmenopausal Women with Decreased Bone Mineral Density. Int J Environ Res Public Health 2020;17(12):4260.doi:10.3390/ijerph17124260). Are there data available on homocysteine levels in the women studied? The lack of this information should be considered among the limitations of the study. It might be interesting to evaluate them, maybe even in a future study. Since hyperomocysteinemia could be a pathogenetic mechanism that may help explain the effects of folate deficiency and MTHFR C677T polymorphism, I suggest that the Authors briefly address this topic in the discussion.

***We thank the reviewer for this suggestion and have edited the limitations section accordingly and will explore the possibilities to examine this in our own future work.***
